# Myogenic Differentiation of Mesenchymal Stem Cells in a Newly Developed Neurotised AV-Loop Model

**DOI:** 10.1155/2013/935046

**Published:** 2013-09-10

**Authors:** Franz F. Bitto, Dorothee Klumpp, Claudia Lange, Anja M. Boos, Andreas Arkudas, Oliver Bleiziffer, Raymund E. Horch, Ulrich Kneser, Justus P. Beier

**Affiliations:** ^1^Department of Plastic and Hand Surgery, University Hospital of Erlangen, 91054 Erlangen, Germany; ^2^Department of Plastic and Hand Surgery, University of BG Trauma Center Ludwigshafen/University of Heidelberg, 67071 Ludwigshafen, Germany; ^3^Interdisciplinary Clinic for Stem Cell Transplantation, University Cancer Center Hamburg (UCCH), 20246 Hamburg, Germany

## Abstract

Generation of axially vascularized muscle tissue constitutes a promising new approach to restoration of damaged muscle tissue. Mesenchymal stemcells (MSC), with their ability to be expanded to large cell numbers without losing their differentiation capacity into the myogenic lineage, could offer a promising cell source to generate neomuscle tissue. In vitro experiments showed that cocultures of primary myoblasts and MSC undergo myogenic differentiation by stimulation with bFGF and dexamethasone. A newly developed AV-Loop model with neurotization was established in this study. It encompasses axial vascularization and the additional implantation of a motor nerve serving as myogenic stimulator. Myoblasts and MSCs were coimplantated in a prevascularized isolation chamber. Cells were differentiated by addition of bFGF and dexamethasone plus implantation of a motor nerve. After 8 weeks, we could observe areas of myogenic differentiation with **α**-sarcomeric actin and MHC expression in the constructs. Quantitative PCR analysis showed an expression of myogenic markers in all specimens. Thus, neurotization and addition of bFGF and dexamethasone allow myogenic differentiation of MSC in an axially vascularized in vivo model for the first time. These findings are a new step towards clinical applicability of skeletal muscle tissue engineering and display its potential for regenerative medicine.

## 1. Introduction/Background

Generation of axially vascularized skeletal muscle tissue is a challenging purpose, promising a new approach to restore damaged skeletal muscle tissue. Diseases, tumour, or trauma can cause a loss of functional skeletal muscle tissue, and solutions to restore these defects are still scarce. Tissue engineering (TE) of skeletal muscle may provide a promising method for this task [1–3]. Mesenchymal stem cells (MSC) seem to be an auspicious cell source because of their ability to differentiate into the myogenic lineage [4–7] *in vitro*, the possibility of allogenic transplantation [8, 9] and their immunomodulatoric potential [10, 11]. *In vitro* experiments showed that the most promising approach in this context is the differentiation by cocultivation with primary myoblasts [4] and additional myogenic stimulation with dexamethasone and basic fibroblast growth factor (bFGF) [12]. The next step towards the final aim of clinical applicability is to translate the *in vitro* results to an *in vivo* setting. Therefore, a matrix providing stability and a biocompatible environment is necessary. This matrix must supply the nutrients to the cells and possibly stimulate them towards myogenic differentiation [13–15]. The implantation of cells in a prevascularized matrix using the rat arteriovenous loop (AV-loop) model is a well-approved strategy in TE to prefabricate tissue constructs [16–19]. Moreover, an additional stimulation in this *in vivo* setting may be desirable. Stimulation by innervation was used in a previous study as first attempt of a physiologic *in vivo* model. However, this single study only allowed the use of a sensoric nerve [20]. Agrawal and Zhang and coworkers study the dependence of motoric innervation in the context of muscle regeneration and motoric neurotization in transplanted muscle flaps [21, 22]. Neurotization can be a stimulus to activate mechanisms of muscle differentiation and contraction. Through the neuron-specific protein agrin, neurons enhance the expression of acetylcholine (ACh) receptors and promote their postsynaptic clustering [23], which presents the first step of formation of neuromuscular junctions and therefore plays a crucial role to enable further differentiation [24]. In the common AV-loop model the possibility of integrating this stimulation by implantation of a motoric nerve was not applicable. A modification of the common AV-loop model in the rat which combines axial vascularization with the implantation of the obturator nerve for motor neurotization can be introduced here as a possibility for motoric innervation in an axially vascularized model for the first time. In the common AV-loop model, this step towards a more physiological setting for skeletal muscle TE is not possible until today. This modified AV-loop model is based on the epigastric vein and the saphenous artery and will therefore be referred to as “EPI-loop” in the following. The first aim of this study is to establish a newly developed AV-model (termed EPI-loop model), which should enable neurotization of an axially vascularized scaffold with a motoric nerve for the first time and comparing it to the common AV-loop technique. The second aim as a step towards *in vivo* application and possible later translation into clinics is the evaluation of the myogenic potential of MSC either implanted alone or together with myoblasts. Development of a new *in vivo* model for tissue engineering of functional skeletal muscle combined with assessment of survival and myogenic differentiation of MSC *in vivo* shall be addressed in this study. 

## 2. Material and Methods

### 2.1. Myoblast Cell Culture

Primary myoblasts were isolated from hind limb muscles of male Lewis rats as described previously [4]. The cell culture medium consisted of DMEM/Ham's F-12 (Biochrom AG, Berlin, Germany) containing 10% FBS and 1% L-glutamine (Gibco/Invitrogen, Auckland, New Zealand). It was changed every second day, and cells were passed when they were subconfluent. Myoblasts of passage 3 were used in this study.

### 2.2. MSC Culture

Mesenchymal stem cells were isolated from male Lewis 1WR2 rats and characterized as described previously [25]. MSC were stably transduced with green fluorescent protein (GFP) for cell labelling, and GFP-positive clones were expanded as described before [25]. All MSC used in this study were of passages 11 and 12. The cell culture medium consisted of DMEM/Ham's F-12 (Biochrom AG, Berlin, Germany) containing 10% FBS and 1% L-glutamine (Gibco/Invitrogen, Auckland, New Zealand).

### 2.3. Predifferentiation Conditions

Cells of each group (either MSC only or cocultivated with primary myoblasts) were expanded up to subconfluency. Before implantation in the *in vivo* setting a predifferentiation period of 5 days was performed. Herefore 5 × 10^5^ myoblasts and 5 × 10^5^ MSC were seeded in a culture flask as coculture. Culture medium was discharged and differentiation medium containing DMEM/Ham's F12, 2% donor horse serum (Biochrom AG, Berlin, Germany), 1% L-glutamine, 1 ng/mL bFGF (Sigma Aldrich, St. Louis, USA), and 0,4 *μ*g/mL dexamethasone (Sigma Aldrich, St. Louis, USA) was added to cell culture dishes as described previously [4]. 

### 2.4. EPI-Loop Model Based on the Epigastric Vein

**Figure 1:**
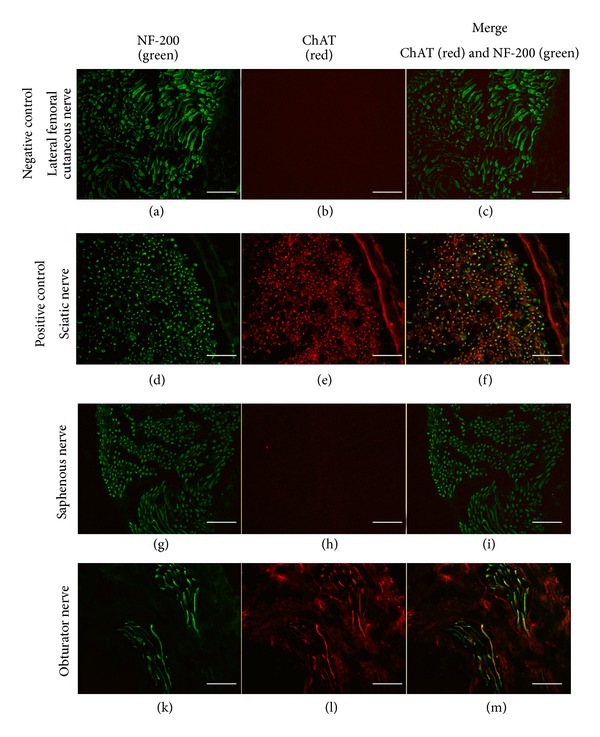
Fluorescence microscopy of NF-200 and ChAT stainings in different nerves. Costaining for neurofilament (NF-200, green) and for Choline-Acetyl-Transferase (ChAT, red). The saphenous nerve (g–i) as well as the lateral femoral cutaneous nerve as negative control for ChAT (a–c) contains no motor neurons and was therefore clearly negative for ChAT. In contrast to the saphenous nerve, the obturator nerve (k–m) as well as the sciatic nerve as positive control (d–f) contains ChAT-positive motor neurons. Scale bar: 50 *μ*m.

All experiments were carried out following the German regulations for the care of laboratory animals at all times. Experiments were approved by the animal care committee of the University of Erlangen and the Government of Mittelfranken, Germany. Male Lewis (Charles River Laboratories, Sulzfeld, Germany) rats were used as *in vivo* model for this study. First, anatomical studies were performed, and the obturator nerve used for neurotization was stained for cholin-acetyltransferase (ChAT) to proof the motoric quality of the implanted nerve. Sciatic nerve served as positive control and lateral femoral cutaneous nerve as negative control. Absence of ChAT in the saphenous nerve shows its valuelessness for motoric neurotization studies (Figure 1). 

Second, the long-term patency of the modified AV-loop model was assessed in a preliminary experiment in 20 rats after 8 weeks *in vivo* to establish the new microsurgical model and optimize the surgical procedure itself. Patency was also assessed 4 days after operation to reassure appreciation for further implantation studies. Isolation chamber was dissected and patency assessed by positive ballooning test in the vein (Table 1).

**Figure 2:**
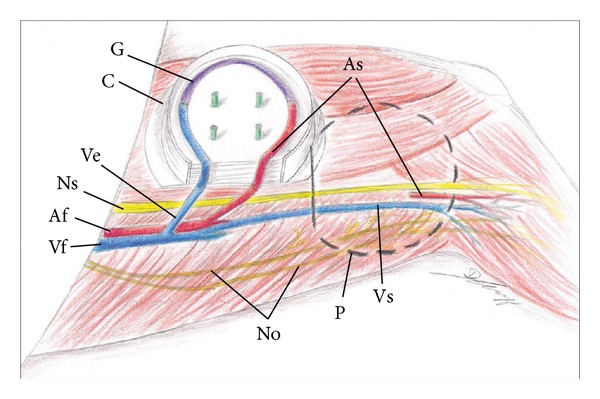
Schematic presentation of the newly developed EPI-loop model. In the common AV-loop model, the position (P) of the isolation chamber directly lies over the path of the obturator nerve (No) which descends the thigh deep inside the adductor muscles. The EPI-loop is based on the saphenous artery (As) and the superficial inferior epigastric vein (Ve)/SIEV and the saphenous vein from the right site as graft (G). In this novel AV-loop model, the isolation chamber (C) can be rotated 90° into the rat's groin and lies parallel to the saphenous nerve (Ns) and vein (Vs). This position enables the dissection of the obturator nerve in a second operation after implantation of the AV-loop into the isolation chamber.

 After establishment of the EPI-loop model as seen in Figure 2, 4 different groups of rats were built with MSC mono- or coimplantation with myoblasts and two different time points (2 or 8 weeks) of explantation. In each group cell implantation and assessment of loop patency were performed after 2 weeks of prevascularization. In these implantation groups 30 animals were operated, and we gained 21 patent loops which were used for the different groups (Co2, Co8 and Mono 8 each with 5 animals, Mono2 with 6 animals) (Table 2).

The modified AV-loop model was based on the superficial inferior epigastric vein connected to the saphenous artery via an interpositional vein graft from the contralateral superficial inferior epigastric vein (SIEV). The patency rate was assessed by dissection of the pedicle and visible pulsation of the arterial pedicle as well as a positive ballooning test in the venous pedicle.

**Figure 3:**
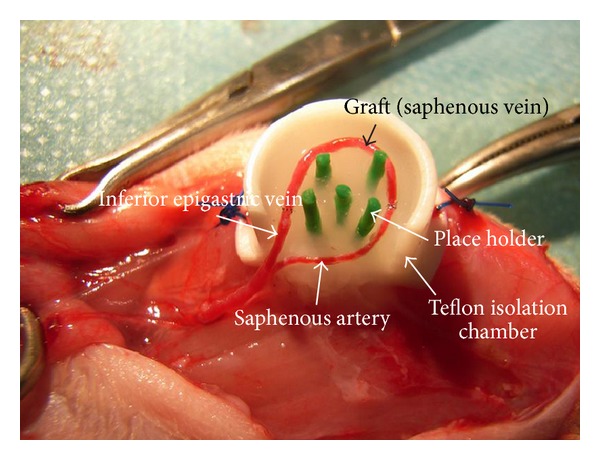
Picture of the implantation of the EPI-loop in the isolation chamber. EPI-loop consisting of the left saphenous artery, superficial inferior epigastric vein (SIEV), and the contralateral SIEV as graft. The loop is embedded in a collagen-fibrin matrix in the Teflon isolation chamber.

Surgery was conducted under inhalation anaesthesia with Isoflurane (Baxter, Unterschleissheim, Germany). Rats left hind limbs were shaved and disinfected accurately. The operation area was covered with surgical drape. The skin was cut in the rat's groin using a T-shaped incision in the left groin and a longitudinal incision in the direction of the inguinal ligament in the right groin (donor site for venous graft). In the left groin, the saphenous artery was dissected up to the saphenofemoral junction under a surgical microscope (Carl Zeiss, Jena, Germany). Then the superficial inferior epigastric vein (SIEV) was dissected from the surrounding fat tissue. The SIEV of the opposite site was harvested as a graft and positioned between the left saphenous artery and left SIEV to gain length and enhance early vessel sprouting as described previously [26]. The AV-loop was generated by anastomosing the graft orthotopically to the left saphenous artery and the left SIEV with 11-0 microsutures (Ethicon, Johnson & Johnson Medical GmbH, Norderstedt, Germany). The generated loop was then implanted into a sterile Teflon isolation chamber with 1 cm in diameter and 0.8 cm in height (medical grade Teflon; P. Greil, Department of Materials Science, Glass and Ceramics, University of Erlangen-Nürnberg). Before implantation of the AV-Loop, a collagen-fibrin matrix was injected in the isolation chamber to gain optimal conditions for prevascularization and padding for the AV-loop. The matrix contained 20 mg/mL fibrinogen in normal saline solution mixed with 4 IU/mL thrombin in 40 mM calcium chloride solution (Tisseel VH/SD, Baxter Healthcare S.A., Wallisellen, Switzerland) and 0,75 mg/mL collagen (rat tail collagen type I, BD Biosciences, Bedford, MA, USA) which was diluted with sterile water and equilibrated to a pH of 7 with NaOH before. All ingredients were mixed to equal parts to a final concentration of 6,66 mg/mL fibrinogen, 1,33 IU/mL thrombin, and 0,25 mg/mL collagen, and 4 spacers were implanted in the centre of the matrix for subsequent nerve implantation and fixation (Figure 3). To enable the dissection of the obturator nerve, the isolation chamber was positioned directly lateral to the saphenofemoral junction and fixed to the underlying quadriceps muscle with 4-0 Prolene sutures (Ethicon, Norderstedt, Germany) at three sides of the isolation chamber. The skin of both sides was closed with resorbable Vicryl 3-0 (Ethicon, Johnson & Johnson Medical GmbH, Norderstedt, Germany). To avoid postsurgical infections, 0,4 mL of an antibiotic (Tardomycel Comp III, Bayer, Leverkusen, Germany) was injected intramuscularly. 1 mL/kg Heparin (80 I.U./kg Liquemin, Ratiopharm, Germany) was injected subcutaneously to prevent thrombosis of the AV-Loop. Animal's behavior and skin closure were controlled every day after implantation.

### 2.5. Neurotization and Cell Implantation

After 14 days of prevascularization, a second surgical procedure was performed to implant the cells and add a motor nerve branch into the chamber. Operations were performed under inhalation anaesthesia as described above. Skin was incised again and the chamber was exposed. After opening the chamber's cap, prevascularization and patency of the AV-loop were assessed by gross examination and ballooning test of the pedicle vein. If both were confirmed, preparation of the motoric obturator nerve was carried out. First, the obturator nerve was dissected from the adductor muscles up to the knee joint. After removal of three spacers within the chamber, the nerve was then implanted into the resulting cavity inside the vascularized matrix and fixed to the fourth spacer using 11-0 microsuture (Ethicon, Johnson & Johnson Medical GmbH, Norderstedt, Germany). 

**Figure 4:**
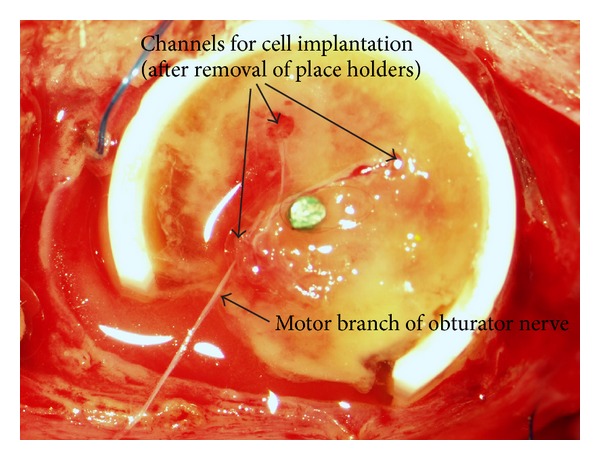
Picture of cell and nerve implantation after 2 weeks of prevascularization. The place holders are already removed, and the motor branch of the obturatoric nerve is implanted and fixed with a stitch around the remaining place holder. After this step, implantation of the cells was carried out.

There were 4 different groups, two with MSC and myoblast cotransplantation and two with MSC only, which were explanted either after 2 weeks or after 8 weeks (Table 1). Cells were treated by predifferentiation with bFGF and dexamethasone as described above. They were prepared for implantation by detaching them with 4 mL Trypsin/EDTA (PAA Laboratories GmbH, Pasching, Austria) from their culture dish. Reaction was stopped with 8 ml of culture medium and a pellet was gained by centrifugation at 1500 rpm for 5 min. Cells were resuspended, counted, and the total number of 6 ×10^6^ cells was centrifuged again. The pellet was resuspended with 80 *μ*L of 20 mg/mL fibrin. To assure the best differentiation conditions 1 ng/mL bFGF (Sigma Aldrich, St. Louis, MO, USA) and 0.4 *μ*g/mL dexamethasone (Sigma Aldrich, St. Louis, MO, USA) were added to the resuspended pellet. After that 80 *μ*L of 4 UI/mL thrombin was added. The cells were implanted by removing the placeholder and inserting cell-matrix-constructs instead. The branches of the motor nerve were inserted in the matrix after cell implantation (Figure 4). After reposition of the cap, the skin was closed with 3-0 Vicryl sutures. 

### 2.6. Explantation

Rats were sacrificed by CO_2_ inhalation for isolation chamber explantation. The specimens were carefully taken out of the chamber and either embedded in Tissue-Tek (Sakura Finetek Europe, Leiden) for preparation of frozen sections or immersed in liquid nitrogen for mRNA isolation. 

### 2.7. Immunohistochemistry

Frozen sections were generated from 5 different areas of each construct with 8 slices per area. They were stained for *α*-sarcomeric actin (Abcam, Cambridge, UK), MyoD1, myogenic enhancer factor 2 (MEF2), and myosin heavy chain (MHC) (all three from Labvision Corp., Hamburg, Germany) according to protocols which were described previously [4]. All primary antibodies were mouseanti-rat IgG1 antibodies. Alexa Fluor 594 goat anti mouse IgG1 (Invitrogen Corp., Karlsruhe, Germany) was used as secondary antibody. Native skeletal muscle tissue from adult Lewis rats served as positive control for all immunocytochemistry stainings and in each group, an isotype control was performed using Mouse IgG1 (BD Biosciences, San Jose, CA, USA) instead of the primary antibody. All probes were analysed and digitally photographed with a fluorescence microscope and camera (Leitz DMRBE, Leica Microsystems, Wetzlar, Germany). No further digital image processing was performed apart from contrast enhancement.

### 2.8. Immunohistochemical Costaining for ChAT and Neurofilament

For ChAT and neurofilament (NF-200) costaining the sciatic nerve was used as positive control and the sensory lateral femoral cutaneous nerve served as negative control. Furthermore, the nerve previously used, which was identified as “femoral nerve” by the authors, in a neurotization model by Messina et al. [20] as well as the obturator nerve were stained to assess the presence of motoric fibers. The sciatic nerve, saphenous nerve, obturator nerve and the lateral femoral cutaneous nerve were identified by typical anatomical landmarks. Serial cryosections of the samples were costained for Choline-Acetyl-Transferase (ChAT) and Neurofilament 200 (NF-200) at a concentration of 1 : 50 each. Slides were incubated with the primary antibody for ChAT (mouse monoclonal IgG; Abcam, Cambridge, MA, USA) overnight and then rinsed with PBS. Afterwards, an AlexaFluor594-labelled secondary antibody against mouse was applied at a concentration of 1 : 200 and incubated for 1 hour (Alexa Fluor 594 goat-anti-mouse IgG1; Invitrogen Corp., Karlsruhe, Germany). After washing, the sections were covered with the primary antibody for NF-200 (neurofilament 200, mouse monoclonal IgG, Clone E14; Sigma-Aldrich, St. Louis, MO, USA) and incubated for 30 min. The sections were rinsed again with PBS, covered with AlexaFluor488-labelled secondary antibody (Alexa Fluor 488 goat-anti-mouse IgG1; Invitrogen Corp., Karlsruhe, Germany) at a dilution of 1 : 1500, and incubated for 40 min. After the last washing step the slides were covered with mounting media Fluoprep (Biomérieux, Marcy l'Etoile, France). The sections were then evaluated and digitally photographed with fluorescence microscopy using the same filter settings and exposure time for all sections.

For methylene blue staining semi-thin sections of specimens explanted 8 weeks after neurotization were dipped into methylene blue solution and washed afterwards thrice.

### 2.9. Quantitative Polymerase Chain Reaction (qPCR)

RNA was isolated from the frozen specimens using the trizol method. The frozen tissue was put into a tube of a ball mill which had been frozen in liquid nitrogen before. Homogenisation was carried out twice. The minced tissue was diluted in 15 mL of Trizol (Invitrogen Corp., Karlsruhe, Germany) and incubated 5 min at room temperature. Then 3 mL of chloroform was added and again incubated for 3 min at room temperature. The specimens were centrifuged with 2500 rpm for 10 min at 4°C. The aqueous supernatant was discharged and again 3 mL of chloroform added. After a centrifugation period of 5 min with 2500 rpm at 4°C, the supernatant was discharged again and 7,5 mL of isopropanol was added. Afterwards, centrifugation with 10000 rpm at 4°C for 10 min was carried out. The gained pellet contained the RNA and was diluted in 1,5 mL of ethanol, dried for 10 min and diluted in 87,5 *μ*L RNAse-free water. According to the manufacturer's protocols, RNA was purified with the RNeasy-kit (Qiagen GmbH, Hilden, Germany). After confirming sufficient RNA concentration and assessment of purity with an Eppendorf Biophotometer (Eppendorf AG, Hamburg, Germany), the probes were reverse-transcribed into cDNA with Omniscript RT-kit, oligo-dT primers for cDNA synthesis, and RNase Inhibitor (Qiagen GmbH, Hilden, Germany). For quantitative PCR, ABsolute QPCR SYBR Green kit was used (Thermo Fisher Scientific, Schwerte, Germany) with a Light Cycler (Bio-Rad iCycler iQ5, Bio-Rad Inc., Hercules, CA, USA). In each group the expression rate of desmin, MyoD1, Myogenin, MEF2, and MHC with GAPDH as endogenous control was analysed. As positive control L6 myogenic cell line, cells (ATCC, Wesel, Germany) were used because of their continuous high expression of myogenic marker RNA. Samples were tested as triplicates and only variations of less then 1.5 threshold cycles were tolerated. Threshold cycles after cycle 35 were defined as invalid. Data evaluation was performed using the ΔΔ*C*_*T*_ method as described previously by Livak and Schmittgen [27].

Primers were as follows:

 Desmin: fwd 5′-ata ccg aca cca gat cca gtc c-3′, rev 5′-tcc ctc atc tgc ctc atc aag g-3′;
 Myogenin: fwd 5′-tga gag aga agg gag gga ac-3′, rev 5′-aca ata cac aaa gca ctg gaa-3′;
 MyoD1: fwd 5′-aga ggg aag gga aga gca gaa g-3′, rev 5′-gca gca gca aca aca acc ag-3′;
 MEF2: fwd 5′-tgc tgc tct cac tgt cac tac-3′, rev 5′-ttc acg act tgg gga cac tg-3′;
 MHC: fwd 5′-tga ctt ctg gca aaa tgc ag-3′, rev 5′-cca aag cga gag gag ttg tc-3′;
 GAPDH: fwd 5′-caa cga ccc ctt cat tga cc-3′, rev 5′-caa cga ccc ctt cat tga cc-3′.

## 3. Results

### 3.1. The EPI-Loop Model in the Rat

The overall patency rate of the 20 EPI-loops was evaluated after four days by a second operation. Isolation chamber was dissected and patency assessed by positive ballooning test in the vein. After 8 weeks, *in vivo *patency was assessed again by direct microscopy of the AV-loop pedicle. Patency rate was 70% (14 patent loops of 20 loops in total). Inside the chamber, vessels were visible on the surface of the matrix and the patent pedicles showed pulsation of the artery and sufficient blood flow with a positive balooning test in the vein. 

The obturator nerve was chosen for neurotization due to its appropriate length (average length was 4.1 cm ± 0.5 cm) which was sufficient to reach the centre of the isolation chamber tension-free in all cases. Although post operative fibrosis of the surgical site was seen in all cases when nerve dissection was done in the second operation, the obturator nerve could be easily identified between the adductor muscles of the thigh. The costaining for NF-200 to identify the neural tissue and for ChAT marking the motor neurons, was positive on sections of the sciatic nerve serving as positive control (Figures 1(d)–1(f)), whereas ChAT was negative in case of the lateral femoral cutaneous nerve as negative control (Figures 1(a)–1(c)). Sections of the saphenous nerve were also negative for ChAT (Figures 1(g)–1(j)). However, the sections of the obturator nerve were clearly ChAT-positive (Figures 1(k)–1(m)).

To enable the dissection of the nerve, the implanted isolation chamber had to be rotated 90° into the groin compared to the common AV-loop model. 

### 3.2. Explantation

All animals tolerated the operations and treatment afterwards well. During explantation, a well vascularised tissue construct was located in the isolation chamber. The pedicle was identified by dissection and cut to harvest the construct. Areas of the tissue constructs showed a typical muscular aspect mostly in the specimens of the 8-week coimplantation group (Co 8). In the other groups, no macroscopic muscular aspects have been detected during explantation.

### 3.3. Immunohistochemistry

**Figure 5:**
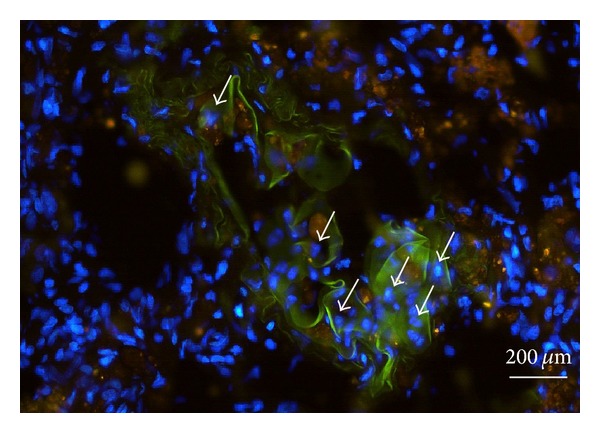
Fluorescence microscopy on GFP-labelled MSC in a harvested construct. After 8 weeks, MSC can still be detected by their green fluorescence. Arrows indicate nuclei of MSC which express GFP (blue = DAPI, green = GFP).

Under fluorescence microscopy survival of MSC implanted in the neurotised AV-Loop model was detected by their green fluorescence based on previous stable transduction with GFP. All specimens showed areas with green fluorescing cells supporting the assumption that MSC survive *in vivo* in the prevascularised, neurotised AV-loop model (Figure 5).

**Figure 6:**
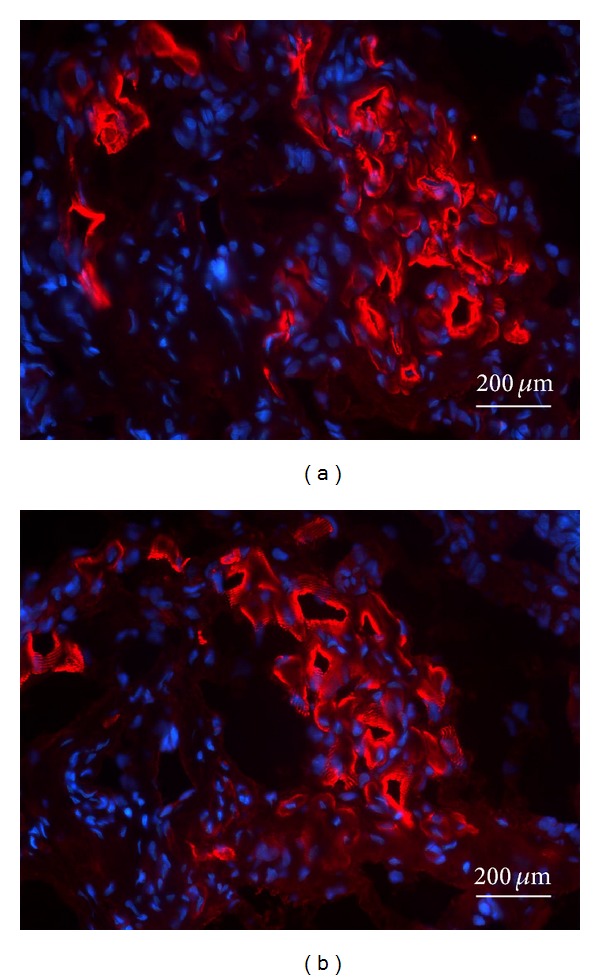
(a) Fluorescence microscopy of *α*-sarcomeric actin staining of a harvested construct. Areas with strong expression of *α*-sarcomeric actin, which is a muscle-specific structure protein for myogenic differentiation, can be observed. Specimen was taken out of the Co8 group (blue = DAPI, red = *α*-sarcomeric actin). (b) Fluorescence microscopy of MCH staining of a harvested construct. The same specimen as Figure 6(a) with a strong expression of MHC as marker for late myogenic differentiation. Muscle-specific cross-striation can be shown here (blue = DAPI, red = MHC).

Myogenic differentiation was observed especially in the cotransplantated group which was explanted after 8 weeks. Here, an advanced myogenic differentiation can be shown by positive staining for *α*-sarcomeric actin (Figure 6(a)) and even for MHC as a marker for late myogenic differentiation (Figure 6(b)). In these areas, cross striation as an indicator for spatial orientation of structural muscle proteins is observed indicating further myogenic differentiation.

**Figure 7:**
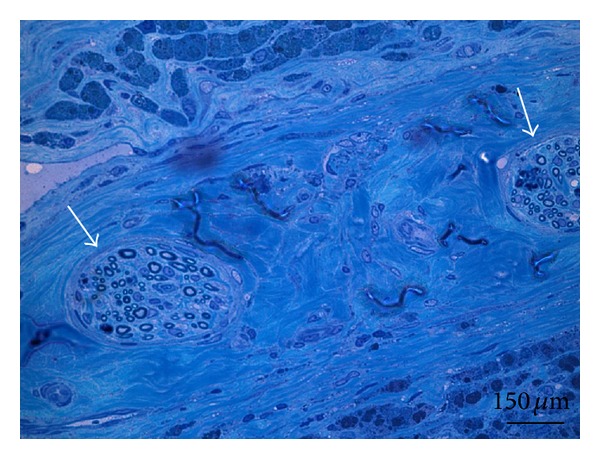
Methylene blue staining of a construct after 8 weeks *in vivo*. Two cross-sections of nerves are clearly visible within the fibrin matrix. Arrows indicate the epineurium of the nerves. Scale bar: 150 *μ*m.

In semithin sections stained with methylene blue, cross sections nerves were found eight weeks after nerve implantation (Figure 7). The implanted fibrin matrix was invaded by host cells. In spite of obvious fibrosis in the surrounding tissue the nerve tissue showed no scar formation or fibrosis eight weeks after neurotization. 

### 3.4. Quantitative Polymerase Chain Reaction

**Figure 8:**
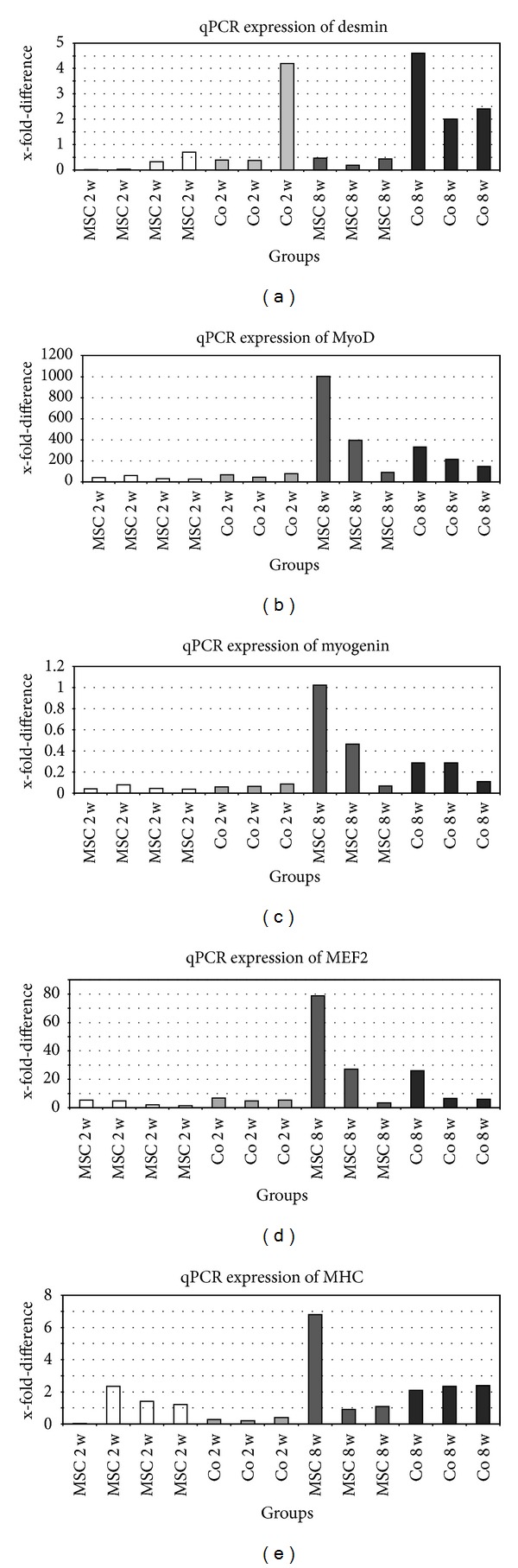
Quantitative polymerase chain reaction results showing expression of desmin, MyoD, Myogenin, MEF2 and MHC. RNA gained from harvested constructs. Results are displayed as x-fold difference compared to a positive control of L6 myogenic cell line cells.

Expression of myogenic markers in the constructs could be observed in all groups. The cycles where the markers got positive in qPCR are presented with their standard deviation. Figures are showing the x-fold difference using the ΔΔ*C*_*T*_ method as described above. The expression of desmin was upregulated mostly in co-implantation groups and the highest expression level was observed in animals of the 8 week co-implantation group Co8 (25,9 ± 0,39 cycles–27,9 ± 0,39 cycles). One animal of the 2-week co-implantation group Co2 showed a high expression (27,57 ± 0,08 cycles) too (Figure 8(a)). 

MyoD1 expression was clearly upregulated in all groups. The highest expression was detected in all three animals of the Co8 group (22,97 ± 0,32 cycles–24,37 ± 0,43 cycles) as well as in two specimens of the Mono8 group (24,63 ± 0,4 cycles; 23,7 ± 2,04 cycles) (Figure 8(b)). Though an upregulation of MyoD1 without the presence of myoblasts can be shown as marker for myogenic potential of MSC. Taken together a high expression of MyoD1 on RNA level can be regarded as evidence for myogenic differentiation of MSC in this long-time *in vivo* setting. 

We could not observe an increase of myogenin expression in any specimen (Figure 8(c)).

The MEF2 expression levels correlate with the results of the expression levels described for MyoD1 (Figure 8(d)). Only one animal in the Co8 group shows a higher expression compared to the 2 week groups (22,57 ± 0,43 cycles). The upregulation of MyoD1 expression in the MSC only long-term group (MSC 8w) shows again evidence for the myogenic potential of MSC as alternative cell source. 

The expression of MHC is clearly enhanced in the Co8 group (26,27 ± 0,41 cycles–27,9 ± 0,33 cycles). Interestingly it is increased in the Mono2 group (21,4 ± 2,92 cycles–22,1 ± 0,38 cycles), too (Figure 8(e)). Taken together the expression of MHC as marker for late myogenic differentiation can be enhanced by the presence of myoblasts as stimulating factor here. These findings confirm our *in vitro* results [4].

## 4. Discussion

TE of skeletal muscle is a promising tool to fabricate tissue constructs which could be used for replacement of soft tissue lost by trauma, cancer, or other acquired or inborn pathologies [2, 28, 29]. The idea of prefabricating transplantable tissue constructs of larger sizes is restricted by disposability of nutrition [30]. Therefore, a transfer from *in vitro* settings to *in vivo* models which include intrinsic vascularization is necessary. A well-established tool to achieve this goal is the AV-loop model [31, 32]. However, to gain functional muscle tissue, besides axial vascularization, an innervation by a motor nerve seems indispensable [22]. In the past mainly myoblasts or satellite cells have been used for skeletal muscle TE, but they showed limited cell survival *in vivo*. MSC are a promising cell source for future applications in this field. Their ability to differentiate into the myogenic lineage combined with their allogeneic transplantability and immunomodulatory potential is their great advantage compared to commonly used primary myoblasts. This study combines optimal differentiation conditions as we described in our previous *in vitro* study where we examined if cell-cell contact in cocultures with primary myoblasts or addition of growth factors only leads to myogenic differentiation in MSC. We could observe hybrid myotube formation with MSC and myoblasts in this study after cell-cell-contact, regarding this setting as the most promising one in terms of myogenic differentiation [4]. Therefore we added myoblasts as stimulator for myogenic differentiation in MSC in this *in vivo* setting. We added nutrition supply by prevascularization [26] and additional stimulation by motoric innervation as an important prerequisite for *in vivo* TE of skeletal muscle. Herein, the obturator nerve appears to be the only nerve in the rat's groin which offers a sufficient length for neurotization as well as the possibility to avoid extended postoperative muscle tissue defects, as in the case of further dorsally located motor nerves like the sciatic nerve.

We suggest that the motoric neurotization should be done at the time of the implantation of muscle progenitor cells after complete vascularization of the matrix to enhance the viability of implanted cells as well as the implanted motor nerve. Sufficient nutrition must be available and can be provided by prevascularization [33]. Most matrices need several weeks of prevascularization before cells can be implanted in a second operation. Therefore, we sought for a loop model which enables a primary as well as a secondary preparation of the motoric nerve so that the model can be used independently from the implanted matrix. However, in the common AV-loop model, the obturator nerve cannot be dissected in a second operation since the isolation chamber is usually fixed at the mid-thigh to the adductorial muscles which contain the obturator nerve. Thus, the chamber had to be rotated 90° into the groin so that the chamber is placed parallel to the saphenous axis, and the adductor muscles with the underlying obturator nerve are not covered by the isolation chamber (Figure 2). To prevent obliteration of the venous vessel, the use of a different vascular axis was necessary. The epigastric vessels, which can be rotated more freely without jeopardizing the blood flow of the loop, can be used as an alternative to the saphenous vessels.

The patency rate of the EPI-loop model after 8 weeks *in vivo* (70% with *n* = 20) was considerably lower than the patency rate of the common AV-loop after 8 weeks *in vivo* (82% with *n* = 11, all operations conducted by the same microsurgeon) [34]. A reason for the lower patency rate could be the learning curve which naturally leads to lower patency rates during the establishment phase of microsurgical models. Thus, the patency rate is still expected to rise. Nevertheless, the patency rate still demonstrates a limitation of this novel AV-loop model.

Survival of MSC in an axially prevascularised setting was demonstrated here for the first time. Previous stable GFP labelling rendered fluorescence microscopy detection of implanted MSC possible. Using techniques to transdifferentiate MSC into the myogenic lineage was adopted from previous *in vitro* studies [4]. A myogenic differentiation up to expression of MEF2 and MHC in several areas shows that this model comprehends great chances for skeletal muscle TE. One limitation of this work is the myogenic gene expression and differentiation to certain constricted areas, while overall myogenic differentiation throughout the whole construct could not be achieved yet. This could be due to temporary limitation of the differentiation conditions. Given the short half-life of dexamethasone and bFGF *in vivo*, its differentiation inducing influence is most likely restricted to the very first days after implantation. Growth factor releasing matrices could be the solution to this problem in future studies. Nanotechnology-based [13, 35, 36] functional matrices could furthermore pose a promising possibility for further studies on skeletal muscle TE [37]. They provide more stability then a fibrin-based matrix. Loss of stability was the reason why constructs were rather small on explantation date in this study. To gain enough RNA for qPCR analysis one whole construct was needed and not all of the 5 five constructs of one group could have been used for qPCR and immunohistochemistry. Therefore, we decided to use 3 constructs of each group for qPCR and 2 for immunohistochemistry.

Another limitation of this work is the lack of a control-group with myoblasts only. The role of MSC in myogenic differentiation might have been estimated more distinctively based on such a group. Therefore this control group will be implemented in future studies.

Insufficient nutrition can be a problem in prevascularized large tissue constructs especially in settings with dense matrices as, for example, electrospun nanofibres. Precultivation with embryonic fibroblast and endothelial cells or human umbilical vein endothelial cells can be a promising approach to gain prevascularization already *in vitro* [38]. Combined with growth factors as vascular endothelial growth factor and fibroblast growth factor 1, this precultivation with myogenic cells can enhance vessel density before implantation of the construct and therefore improve cell survival for further differentiation [39].

However, signs of myogenic differentiation on mRNA level were shown here by evaluation with qPCR, providing evidence for myogenic differentiation in all specimens, while differentiation up to myogenic gene expression on protein level was mostly seen in the Co8 animals. The qPCR as a very sensitive method revealed early signs of myogenic differentiation of MSC in the newly developed EPI-loop model. However, these signs were not supported by positive MSC expression of myogenic markers on protein level. Possibly improvement of sensitivity as well as GFP co-staining in for histological analyses as well as more serial sections may give further insight into MSC differentiation and coculture interaction during future *in vivo* studies, as demonstrated by our group *in vitro* before. Taken together, first signs for myogenic differentiation of MSC in a prevascularized and neurotised *in vivo* setting were described here for the first time. Cross-sections of nerve tissue were found eight weeks after nerve implantation. The nerve tissue was healthy and showed no sign of fibrosis or apoptosis in methylene blue staining. This was regarded as adequate proof for the survival of the implanted nerve tissue. In further studies we plan to assess the establishment of motor endplates to show the myogenic stimulus of neurotisation [40].

New options, like injection of micro-RNAs [41] or predifferentiation of larger constructs in a bioreactor [42–44] need to be considered as additional possibilities to achieve a more constant differentiation for *in vivo* applications. 

Adipose derived stem cells can be discussed as alternative stem cell-based cell source for muscle tissue engineering as well [45]. Their abilities are already well known regarding osteogenic and chondrogenic differentiation capacity, and they seem to be an equal cell source compared to bone marrow-derived MSC in these applications [46]. 

A new microsurgical model for axial vascularization is presented here for the first time as an important step towards a physiological *in vivo* approach to TE of skeletal muscle. Survival of MSC in an axially vascularized in vivo model was shown here for the first time. Early signs of myogenic differentiation of MSC *in vivo* were observed. 

Further studies with optimization of the differentiation conditions, assurance of continuous motor innervation and implementation of functional nanoscaffolds may further increase the potential of this approach. 

## 5. Conclusion

Establishing an adequate *in vivo* model is still a major challenge for TE of functional skeletal muscle. Parameters such as the vascularization, innervation, matrix constitution, and myogenic cell differentiation play an important role and need to be integrated to constitute a promising model. The new modification of the well-established AV-loop model by integration of a motor nerve towards the EPI-loop model is a challenging microsurgical approach. The EPI-loop model is based on both superficial inferior epigastric veins (SIEV) and the saphenous artery for axial vascularization, thereby enabling later microsurgical transplantation, while at the same time, motoric neurotization is facilitated through obturator nerve implantation. Addition of MSC as a myogenic cell source opens a new route towards the aim of clinical application. We could show that MSC survive in this *in vivo* model and that myogenic differentiation is achieved upon growth factor application and motor nerve stimulation. MSC are an important alternative cell source, but better differentiation stimuli have to be found to efficiently use this source. Upon further investigations, this study could be a promising step towards TE of functional skeletal muscle tissue.

## Figures and Tables

**Table 1:** Preliminary experiment and implantation experiment with a number of operated animals and patency rates.

Features	Experiment	
	Preliminary experiment	Implantation experiment
Operated animals	20	30
Patent loops	14	21
Patency rate	70%	70%

**Table 2:** Different subgroups with cell types and *in vivo* periods of the implantation group. Number of specimens which were taken for the different methods (qPCR and IHC).

Features	Groups (cells)			
	Mono2 (MSC only)	Mono8 (MSC only)	Co2 (myoblasts + MSC)	Co8 (myoblasts + MSC)
Implantation period (after prevascularization)	2 weeks	8 weeks	2 weeks	8 weeks
Number of animals	6	5	5	5
Animals for qPCR	4	3	3	3
Animals for IHC	2	2	2	2
